# Spatial cell graph analysis reveals skin tissue organization characteristic for cutaneous T cell lymphoma

**DOI:** 10.1038/s41540-024-00474-x

**Published:** 2024-12-02

**Authors:** Suryadipto Sarkar, Anna Möller, Anne Hartebrodt, Michael Erdmann, Christian Ostalecki, Andreas Baur, David B. Blumenthal

**Affiliations:** 1 Biomedical Network Science Lab, Department Artificial Intelligence in Biomedical Engineering (AIBE), Friedrich-Alexander-Universität Erlangen-Nürnberg (FAU), Erlangen, Germany; 2 Department of Dermatology, Uniklinikum Erlangen, Deutsches Zentrum Immuntherapie (DZI), Comprehensive Cancer Center Erlangen-European Metropolitan Area of Nuremberg (CCC ER-EMN), Friedrich-Alexander-Universität Erlangen-Nürnberg (FAU), Erlangen, Germany

**Keywords:** Single-cell imaging, Cancer, Computational biology and bioinformatics

## Abstract

Cutaneous T-cell lymphomas (CTCLs) are non-Hodgkin lymphomas caused by malignant T cells which migrate to the skin and lead to rash-like lesions which can be difficult to distinguish from inflammatory skin conditions like atopic dermatitis (AD) and psoriasis (PSO). To characterize CTCL in comparison to these differential diagnoses, we carried out multi-antigen imaging on 69 skin tissue samples (21 CTCL, 23 AD, 25 PSO). The resulting protein abundance maps were then analyzed via scoring functions to quantify the heterogeneity of the individual cells’ neighborhoods within spatial graphs inferred from the cells’ positions in the tissue samples. Our analyses reveal characteristic patterns of skin tissue organization in CTCL as compared to AD and PSO, including a combination of increased local entropy and egophily in T-cell neighborhoods. These results could not only pave the way for high-precision diagnosis of CTCL, but may also facilitate further insights into cellular disease mechanisms.

## Introduction

Cutaneous T-cell lymphomas (CTCL) present in the form of erythematous lesions, eruptions or patches on the skin. These lesions can both clinically and histologically resemble other non-cancerous inflammatory dermatological conditions, including atopic dermatitis (AD) and psoriasis (PSO)^1–4^, making diagnosis of CTCL challenging. Since CTCL needs to be treated thoroughly early on^5–9^, this is a major clinical problem. Techniques to reliably distinguish CTCL from the mimicking conditions AD and PSO therefore hold the promise to improve patient care in CTCL.

While several studies have explored the spatial heterogeneity of the tumor microenvironment in CTCL and its potential relevance in prognosis^10,11^, a systematic quantification of spatial tissue heterogeneity in the context of CTCL does not exist. This prompted us to generate imaging-based spatially resolved protein abundance maps of skin tissue samples from CTCL, AD, and PSO patients treated at the University Hospital Erlangen, using multi-epitope ligand cartography (MELC)^12,13^. Based on a visual assessment of the images, tissue organization was indeed altered in CTCL compared to AD and PSO samples. To quantify and objectivize this first subjective impression, we used the popular Squidpy package^14^ to generate graph representations of our data, where nodes are cells annotated with their cell type and edges encode spatial vicinity.

Since analysis with existing techniques available in Squidpy revealed only few differences, we developed a Python package called SHouT (short for “spatial heterogeneity quantification tool”, available at https://github.com/bionetslab/SHouT), which allows to quantify tissue heterogeneity based on spatial cell graphs. Relying on well-established concepts such as Shannon’s entropy^15^ which have been successfully used for spatial heterogeneity quantification in medical imaging^16–18^ and single-cell omics data^19–21^, SHouT provides fast and user-friendly implementations of six graph-based scores to characterize tissue organization in spatial omics data. SHouT revealed clear CTCL-specific characteristics of tissue organization, including higher mixing of cells of different types in the vicinity of T cells in CTCL as compared to AD and PSO. Randomization tests based on label permutation and subsampling verified the robustness of these findings.

## Results

### Overview of study design

**Fig. 1:**
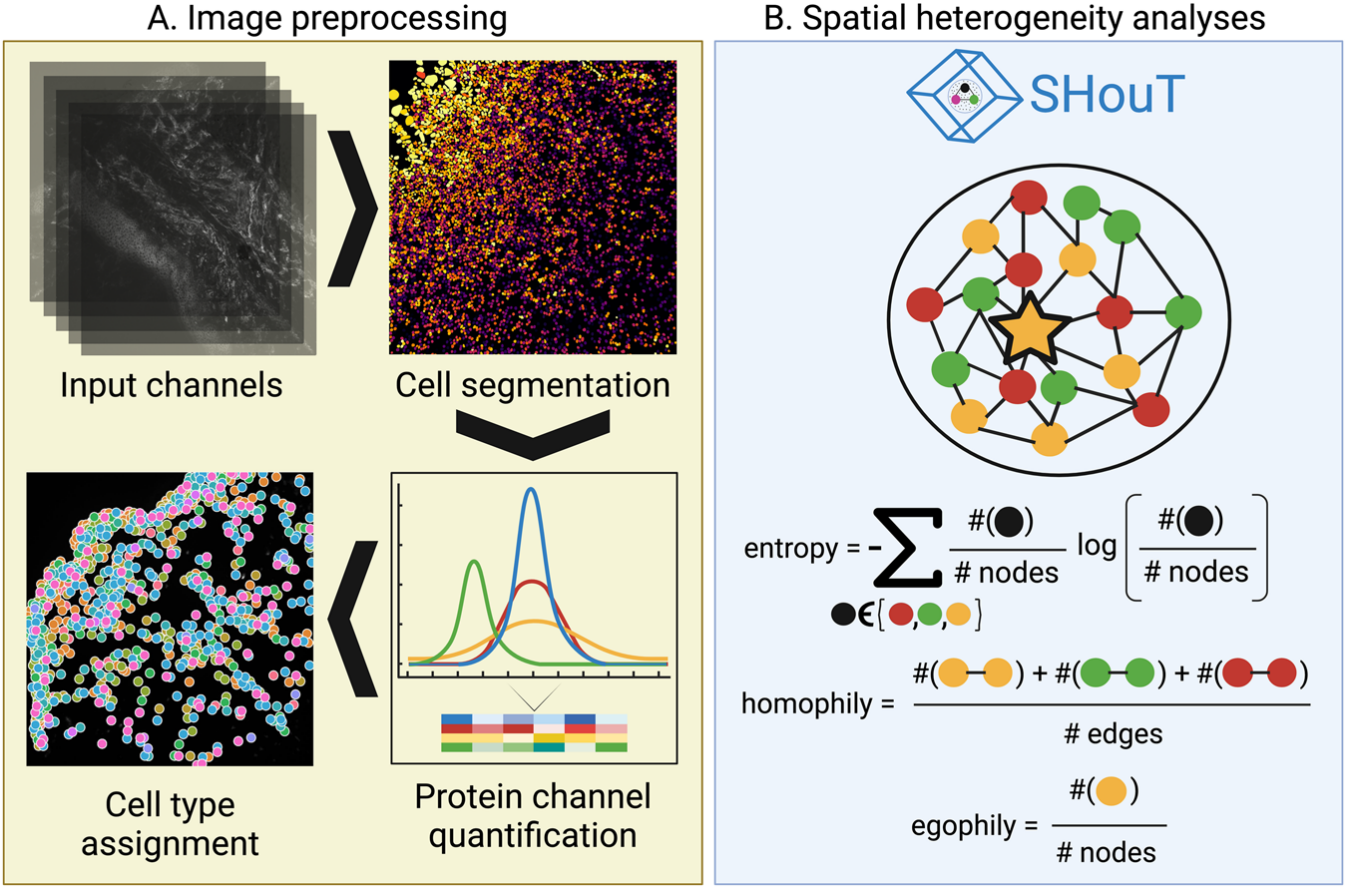
Overview of our analyses. **A** We generated multi-antigen images for skin tissue samples from CTCL, AD, and PSO patients. Subsequently, images were pre-processed via cell segmentation, cell-level protein abundance quantification, and cell-type assignment. **B** We then computed spatial graph representations for all samples, which we analyzed using Squidpy, Leibovici’s entropy, modularity, as well as different heterogeneity scores implemented in our Python package SHouT: local and global entropy, local and global homophily, and egophily.

Figure 1 provides an overview of our work (see “Methods” for details): Using MELC, we generated spatial protein abundance maps for 69 skin tissue samples (21 CTCL, 23 AD, 25 PSO). With this, we obtained images of resolution 2018 × 2018 pixels for at least 35 protein channels per sample, leading to over 140 million pixel values. We then carried out cell segmentation for all images, using the propidium iodide and CD45 channels as markers for the nucleus and cell membrane, respectively. Upon manual inspection of the segmentation results, one CTCL sample was excluded due to insufficient segmentation quality, leaving us with a cohort of 68 patients in total. Subsequently, adaptive thresholding was used to quantify protein abundances within the individual cells, and cell types were assigned via a rule-based marker gene approach (Supplementary Fig. 1), using skin tissue single-cell RNA sequencing (scRNA-seq) data from the Human Protein Atlas (HPA)^22^ as reference. We then projected the cell-type labels back on the 2018 × 2018 image grid, and quantified heterogeneity of cell-type co-occurrence patterns in the individual samples via Leibovici’s entropy^23^ (using the leibovici function from the geoentropy package available at https://github.com/maxkryschi/geoentropy).

Quantifying spatial heterogeneity of spatial omics data with methods such as Leibovici’s entropy that operate directly on the image space is straightforward but has the disadvantage that pixels, i.e. entities without direct biological meaning, are the primary units of analysis. To also enable cell-based spatial heterogeneity quantification, we therefore computed a spatial graph for each sample, where two cells are connected by an edge if they are adjacent to each other in the spatial map. The spatial graphs were then analyzed with Squidpy’s centrality_scores and nhood_enrichment functions, which compute the degree and closeness centralities of the individual cells within the spatial graphs and enrichment of cell-type co-occurrence within one-hop spatial neighborhoods, and by computing the modularity^24^ of the node partitions induced by the assigned cell types (using the modularity function of NetworkX^25^). We selected these scores because they are readily available in the Python packages Squidpy and NetworkX, which are very widely used in the field of spatial omics analysis. However, it is important to note that none of these scores was originally proposed as a score to distinguish between samples from different conditions (for instance, the primary purpose of the neighborhood enrichment method is to mine spatial omics data for cell communication events).

In addition to relying on these existing tools, we analyzed the cell graphs with our novel Python package SHouT, which we developed with the task of downstream differential analysis in mind. SHouT provides two sample-level scores that quantify overall tissue heterogeneity:

- The edge-based *global homophily* score represents the fraction of edges in the spatial graph that connect cells of the same type. A high score indicates low overall heterogeneity.
- The node-based *global entropy* score represents how well-balanced the numbers of cells of each cell type are throughout the network. A high score indicates high overall heterogeneity.

Moreover, SHouT provides three cell-level scores that quantify tissue heterogeneity within the *r*-hop neighborhood of an individual cell *c* in the spatial graphs (Fig. 1B):

- The edge-based *local homophily* score represents the fraction of edges in the *r*-hop neighborhood of *c* that connect cells of the same type. A high score indicates low heterogeneity in the tissue region surrounding cell *c*.
- The node-based *local entropy* score represents how well-balanced the numbers of cells of each cell type are in the *r*-hop neighborhood of *c*. A high score indicates high heterogeneity in the tissue region surrounding cell *c*.
- The node-based *egophily* score represents the fraction of cells within the *r*-hop neighborhood of *c* that have the same cell type as *c*. A high score indicates low heterogeneity in the tissue region surrounding cell *c*.

By specifying the radius *r*, the user can select the desired granularity of the cell-level scores. With *r* = 1, only cells in the immediate vicinity of *c* are considered. On the other extreme, setting *r* to the diameter of the spatial graph renders local homophily and entropy equivalent to global homophily and entropy, respectively. Setting *r* to intermediate values allows the quantification of mesoscale patterns in the spatial graphs.

**Fig. 2:**
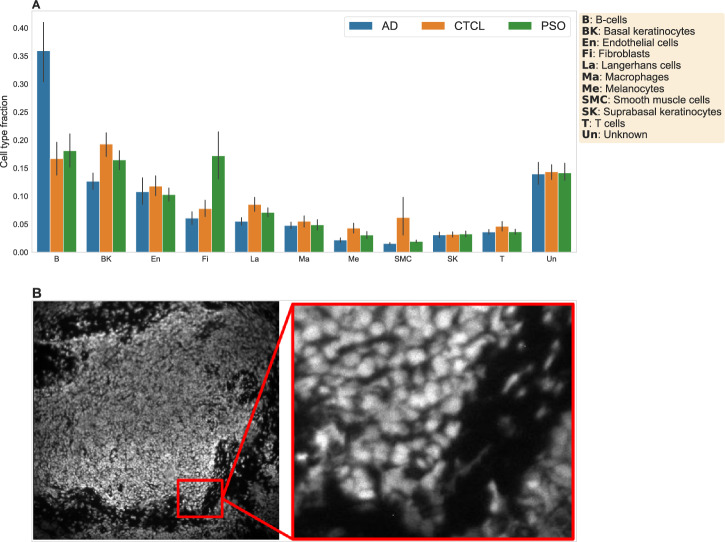
Summary of the multi-antigen imaging dataset used for this study. **A** Mean cell-type fractions per condition, across all of the samples. **B** The propidium iodide channel used for cell segmentation for one sample. Left: Compressed image of the entire tissue section. Right: Magnified view representing the actual resolution used for cell segmentation.

Figure 2 provides a high-level overview of the dataset. Figure 2A shows the average cell-type composition stratified by sample type (see Supplementary Fig. 2 for underlying distributions of sample-specific cell-type fractions and Supplementary Fig. 3 for a joint t-SNE projection of the protein expression data of all 68 patients, colored by cell type). With the exception of an increased abundance of endothelial cells in AD samples and increased abundance of macrophages in PSO samples, we did not observe strong differences in cell-type composition between the three conditions, emphasizing the need for more in-depth analyses. Figure 2B shows the propidium iodide channel used for cell nucleus identification for one sample.

### Heterogeneity analysis reveals CTCL-specific patterns in the neighborhoods of T cells and basal keratinocytes

**Fig. 3:**
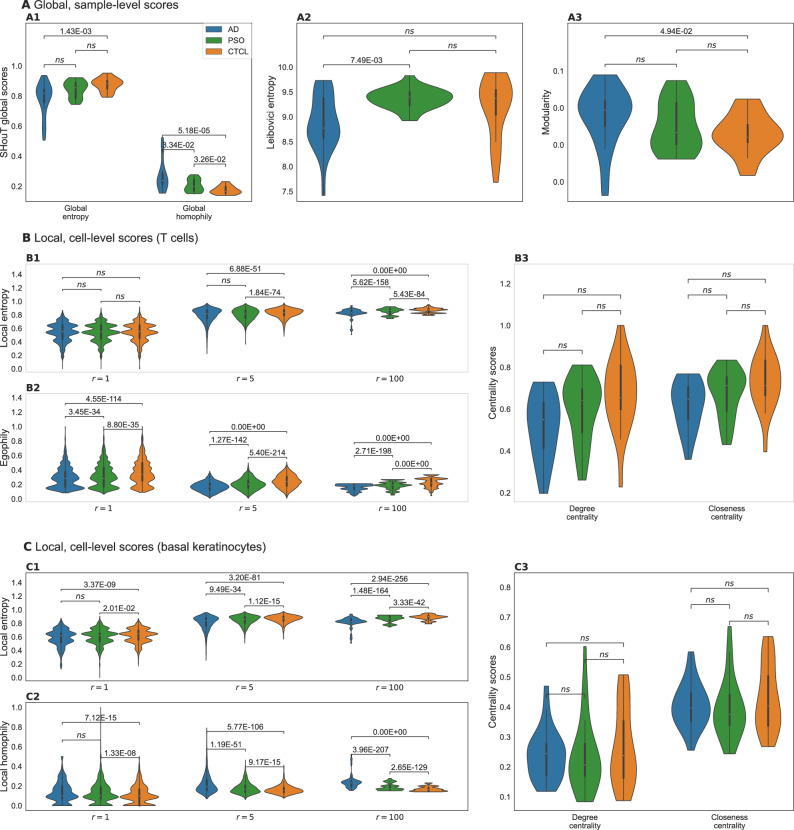
Results of spatial analyses of AD, PSO, and CTCL samples with adjusted MWU *P* values. The most important differences between CTCL samples as compared to AD or PSO are: elevated local entropy and egophily scores for neighborhoods of T cells; and elevated local entropy and local homophily scores for neighborhoods of basal keratinocytes. **A** Distributions of global (i.e., sample-level) scores for the different conditions. **B** Distributions of local (i.e., cell-level) scores for all T cells from samples from the different conditions. **C** Distributions of local scores for all basal keratinocytes from samples from the different conditions.

Figure 3 shows the results of analyzing the spatial graphs with SHouT, Leibovoci entropy, modularity, and Squidpy’s centrality_scores function (see Supplementary Fig. 10 for the results obtained with the nhood_enrichment function). All shown *P* values were computed with the two-sided Mann–Whitney *U* (MWU) test and were Bonferroni-corrected with respect to the number of tests per score type (neighborhood enrichment scores: number of condition pairs × number of cell-type pairs; centrality scores, network modularity, Leibocovi centrality, and all global SHouT scores: number of condition pairs; local heterogeneity SHouT scores: number of condition pairs × number of cell types × number of tested radii (10)).

The two global heterogeneity scores show that overall tissue heterogeneity is highest in CTCL (lowest global homophily, largest global entropy), followed by PSO and AD (Fig. 3A1). This is supported by the results obtained with Leibovici centrality (Fig. 3A2, largest values for CTCL, indicating higher heterogeneity) and network modularity (Fig. 3A3, lowest values for CTCL, indicating decreased clustering of cells of the same type). However, of all four global heterogeneity measures, only the differences in global homophily reach statistical significance for both the comparison between CTCL and PSO and the comparison between CTCL and AD.

When focusing on T cells (Fig. 3B), we observe significantly elevated local entropy (Fig. 3B1) and egophily (Fig. 3B2) scores in CTCL. That is, in skin samples from CTCL patients, T cells tend to cluster together (unsurprisingly, since the tumor cells are T cells) and at the same time are surrounded by tissue that exhibits a higher mixing of cell types than the spatial neighborhoods of T cells in AD or PSO (increased local entropy). Interestingly, the differences are more pronounced and the score distributions are smoother for radius *r* = 5 than for *r* = 1, thereby highlighting the importance of incorporating *r*-hop neighborhoods into SHouT’s local heterogeneity scores. At radius *r* = 100, the score distributions become less smooth, but nonetheless maintain the pronounced differences between the conditions (see Supplementary Figs. 4–6 for additional results with *r* = 5 for all cell types; and Supplementary Fig. 7 for distributions of local SHouT scores across radii *r* ∈ {1, 2, 3, 4, 5, 10, 20, 50, 100, 500} in T-cell neighborhoods).

In contrast to SHouT, Squidpy’s centrality_scores function did not reveal significant differences between the three conditions (Fig. 3B3, see Supplementary Fig. 9 for additional results corresponding to all cell types). Neighborhood enrichment analysis with Squidpy’s nhood_enrichment function (Supplementary Fig. 10) did reveal some differences in co-occurrence between T cells and, respectively, macrophages, fibroblasts, and basal keratinocytes. However, the observed differences are much smaller than for local entropy and homophily, do not reach statistical significance after multiple testing correction, and no clear picture emerges that would allow to robustly distinguish CTCL samples from AD and PSO samples based on neighborhood enrichment scores of T cells.

We obtained highly significant differences in SHouT’s local heterogeneity scores also for another cell type besides T cells, namely, basal keratinocytes (Fig. 3C, see Supplementary Fig. 8 for distributions of local SHouT scores across radii *r* ∈ {1, 2, 3, 4, 5, 10, 20, 50, 100, 500} in basal keratinocyte neighborhoods). Like for T cells, local entropy is increased in CTCL in comparison to AD and PSO (Fig. 3C1), i.e., tissue in the vicinity of basal keratinocytes exhibits a higher cell-type mixing in CTCL than in the two other conditions. Moreover, and in contrast to the results for T cells, we simultaneously observe elevated local homophily scores for basal keratinocytes (Fig. 3C2). That is, tissue in the vicinity of basal keratinocytes exhibits a higher co-localization of cells of the same type in CTCL than in PSO or AD (increased local homophily), even though the cell-type heterogeneity is increased (increased local entropy). This shows that the seemingly similar local entropy and local homophily scores indeed quantify distinct properties of spatial tissue organization. As for T cells, the differences between the three conditions are more pronounced for *r* = 5 than for *r* = 1. For larger radii including *r* = 100, the differences remain highly significant, but the score distributions again become noisier than for *r* = 5.

Squidpy’s centrality_scores function did not reveal significant differences related to basal keratinocytes (Fig. 3C3). Neighborhood enrichment analysis, however, showed that in CTCL samples, the occurrence of macrophages, melanocytes, smooth muscle cells, suprabasal keratinocytes, and Langerhans cells is increased in the vicinity of basal keratinocytes (Supplementary Fig. 10), although results do not reach statistical significance after multiple testing correction.

### The identified CTCL-specific patterns are robust to label permutation and subsampling

**Fig. 4:**
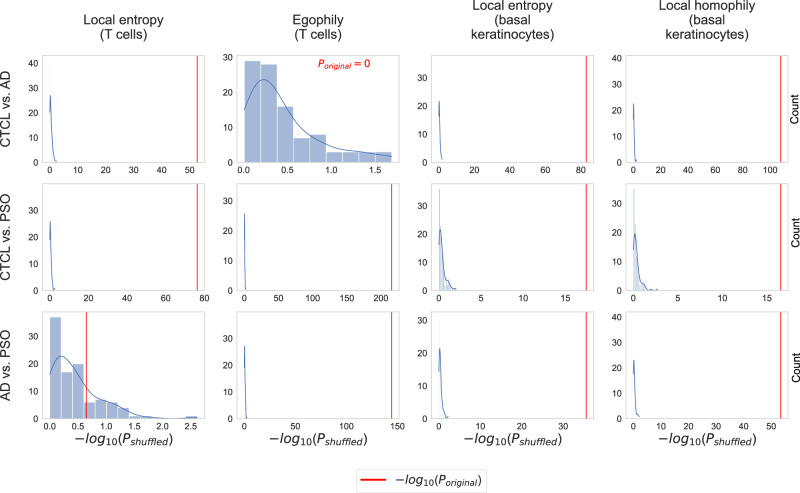
Results of permutation tests to assess the robustness of the differences in the local heterogeneity scores with radius *r* = 5 shown in Fig. 3. All *P* values were computed with the MWU test. Unlike for the *P* values shown in Fig. 3, we did not apply Bonferroni correction to adjust for multiple testing (since we are comparing *P* values, adjusting for multiple testing leaves the results invariant).

We carried out permutation tests to assess the reliability of the identified differences in the local heterogeneity scores with radius *r* = 5 shown in Fig. 3. Specifically, we shuffled the condition labels across all 68 samples and then used the MWU test to compare the local heterogeneity scores between the obtained randomized conditions. We repeated this for 100 iterations, leading to 100 *P* values per cell type and condition pair. Figure 4 shows the resulting *P* value distributions (histograms), together with the *P* values for the original condition labels (red lines). For all eleven combinations of scores and condition pairs where results are significant for the original, unshuffled condition labels (all plots in Fig. 4, except for the one at the bottom left), the *P* values obtained for the original condition labels are much smaller than those obtained for the shuffled labels. This indicates that the heterogeneity scores indeed reveal robust differences between CTCL, AD, and PSO.

**Fig. 5:**
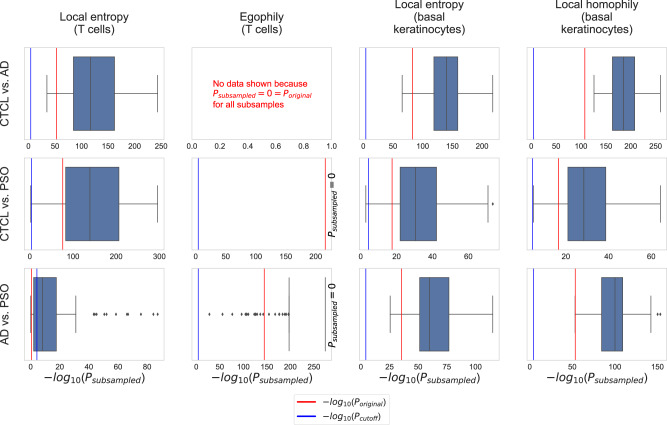
Results of subsampling tests to assess the robustness of the differences in the local heterogeneity scores with radius *r* = 5 shown in Fig. 3. All *P* values were computed with the MWU test. The significance cutoff is Bonferroni-adjusted, i.e., *P*_cutoff_ = 0.05/(number of condition pairs × number of cell types × number of tested radii) = 0.05/(3 × 11 × 10). *P* values are non-adjusted.

Moreover, we carried out subsampling to assess if the identified CTCL-specific tissue organization patterns are robust to varying compositions of the CTCL, AD, and PSO cohorts. Specifically, we subsampled 15 samples for each of the three conditions. Subsequently, for each condition pair, we computed MWU *P* values based on the SHouT’s local heterogeneity scores (with *r* = 5) for T cells and basal keratinocytes, using only the samples from the subsampled patients. We repeated this 100 times, leading to 100 MWU *P* values for each condition pair, considered cell type, and SHouT score. The resulting *P* value distributions (boxplots) are shown in Fig. 5, together with the *P* values obtained when using all samples (red lines) and the Bonferonni-corrected significance cutoff *P*_cutoff_ = 0.05/(number of condition pairs × number of cell types × number of tested radii)(blue lines). For all cases where the original *P* values are significant (red lines to the right of blue lines), all or the vast majority of the *P* values obtained upon subsampling are significant, too. This shows that SHouT reveals CTCL-specific tissue patterns that are robust to subsampling.

### SHouT scales to samples with large numbers of cells

**Fig. 6:**
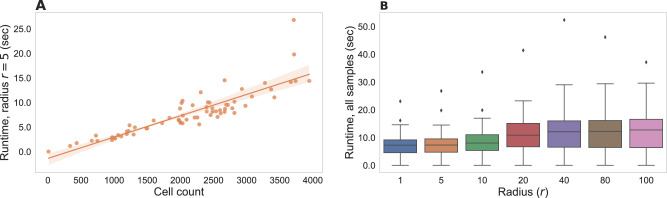
Results of scalability tests for our Python package SHouT. Runtime measurements include all subroutines detailed in the “Quantification of spatial tissue heterogeneity with SHouT” subsection of the “Methods”: construction of the spatial neighborhood graph *G* = (*V*, *E*, *λ*_*V*_), computation of the two global scores *H*(*G*) and *h*(*G*), and computation of the three local scores *H*_*r*_(*c*), *h*_*r*_(*c*), and *e*_*r*_(*c*) for all cells *c* ∈ *V* and a fixed radius *r*. To ensure a stable execution environment, tests were run on a Linux compute server with 500 GB of main memory and four AMD EPYC 7402 24-core processors with 1.5 Ghz (without using multi-threading). However, SHouT does not require such large-scale resources and can be run on a standard laptop. **A** Runtimes with a fixed radius *r* = 5 for all samples in our dataset. **B** Runtime distributions for varying radii across all samples in our dataset.

To ensure usability, runtime efficiency is an important property of data-centric methods for the analysis of biomedical data. We therefore systematically tested SHouT with respect to its runtime requirements when run on samples with varying numbers of cells or when run with varying radii. The results are shown in Fig. 6. We observe that SHouT scales linearly with the numbers of cells per sample, achieving runtimes of little more than a minute even for the samples with the highest cell counts in our dataset (Fig. 6A). Increasing the radius *r* only marginally increases the runtime, showing that SHouT’s heterogeneity scores can be computed efficiently independently of the choice of *r* (Fig. 6B).

## Discussion

Our analyses identified CTCL-specific patterns of tissue organization as compared to PSO and AD in the vicinity of T cells and basal keratinocytes. Since CTCL is a T-cell malignancy, observing characteristic patterns in the vicinity of T cells is not too surprising. In fact, existing studies suggest that malignant T cells and their cross-talk with other cells induce disorganization in the epidermal architecture^26–28^, which is well aligned with our findings. Also, our results for basal keratinocytes are plausible in the light of the literature. For instance, several studies have identified hyperproliferation and/or de-differentiation of keratinocytes in CTCL^27,29,30^.

An important aspect of our findings is that the SHouT scores underlying our results are (1) purely quantitative, (2) interpretable by design (each SHouT score has a natural interpretation that can be explained with few sentences), and (3) deterministically computed (computing the SHouT scores does not involve randomized subroutines). Their purely quantitative nature sets our SHouT scores apart from the current standard of care in CTCL diagnosis, which is typically based on (subjective) expert opinions. Their interpretability is a decisive advantage over predictions provided by deep learning models, which are often perceived as black boxes^31^. The fact that SHouT is deterministic ensures that it consistently computes the same results when run several times on equivalent input—unlike many other methods in data-centric biomedicine^32,33^. Together, these three properties make the SHouT scores ideal ingredients for potential future biomarkers based on spatial omics data, both in the CTCL use case presented here and beyond. Therefore, we are convinced that SHouT will be of value for the analysis of spatial omics data also beyond this study, complementing existing toolboxes such as Squidpy.

However, several limitations of our study have to be addressed before translation into clinical care may become feasible. First and foremost, the MELC technology used to generate the data underlying this study is highly non-standardized (only few prototypes exist worldwide, two of them at UKER). This had strong implications for the data analysis pipeline used for this study, where we had to resort to customized solutions for almost all pre-processing steps (cell segmentation, protein abundance quantification, cell-type assignment). While we are confident that the methodological choices we made for individual steps in our pipeline are adequate for the data, they still introduce a certain amount of contingency which may distort the results of our analyses in a hard-to-control way. Before translation into clinical care can become an option, it would hence be important to see if our results can be reproduced for spatial omics generated with more established technologies (e.g., MERFISH^34^ or MIBI^35^), for which validated pre-processing pipelines are available.

## Methods

### Sample collection and multi-antigen imaging

In total, 69 skin tissue samples (21 CTCL, 23 AD, 25 PSO) from a total of 27 treated patients (8 CTCL, 7 AD, 12 PSO) were collected at the University Hospital Erlangen. The study has been approved by the Ethics Committee of the Medical Faculty of the Friedrich-Alexander-Universität Erlangen-Nürnberg (approval date: July 5, 2023; approval number: 23-132-B). We used the MELC technology^12,13,36^ to generate multi-antigen imaging data. MELC efficiently combines seamless assimilation of molecular and anatomical information in situ by employing a cyclic process of three steps: (1) protein-specific fluorescent antigen staining, (2) imaging, and (3) photobleaching. The workflow is completely automated, performing multi-antigen imaging on a fixed and mounted tissue or cell sample, without the requirement of any human involvement. Thanks to this automated workflow, post imaging, the individual protein channels can be mapped into one consolidated tissue map whilst preserving spatial information. In order to assemble a set of antibodies with a strong and specific staining pattern, we screened over 500 antibodies on CTCL tissue. This unbiased approach yielded 36 antibodies (Supplementary Table 1).

### Cell segmentation and protein abundance quantification

Owing to the properties of DNA- and histone-binding, propidium iodide is pervasively used to stain cell nuclei in fluorescent microscopy^37^. We therefore used the propidium iodide channel for cell nucleus segmentation, relying on a pre-trained model provided by the stardist.models.StarDist2D function of the popular cell detection library StarDist^38,39^.

For cell membrane segmentation, we used the channel for the transmembrane protein tyrosine phosphate (CD45), which is not only present in all nucleated hematopoietic cells but is also one of the most commonly found membrane proteins in such cells^40^. However, owing to the often non-convex shape of cell membranes as opposed to cell nuclei in our dataset, the StarDist model under-segments the cytoplasm. As a workaround, for all nuclei where the StarDist model managed to automatically identify the cell membrane, we calculated an average ratio between the radii of cells and nuclei. We then used this average ratio to draw a circle around each of the segmented nuclei for which the StarDist model did not identify a cell membrane. If two such circles overlapped, pixels within the intersection were assigned to the closest nucleus, thereby ensuring that all cell segments were pairwise disjoint. All segmentation results were manually inspected by a histopathology expert, and one CTCL sample was excluded due to insufficient segmentation quality.

Transitioning from pixel intensity to cell-level protein abundances was challenging due to the following factors: (1) MELC images are especially susceptible to salt-and-pepper noise. (2) Different protein channels have different intensity levels. (3) Even within the same channel, different regions often have different levels of intensity. Because of these factors, there is no single mapping which would allow to uniformly compute cell-level protein abundance scores based on pixel intensities across all samples and sample regions. We therefore made use of adaptive thresholding which helps circumvent this issue by breaking the image into smaller windows comprising fewer pixels, subsequently computing a different threshold for every window. Specifically, we used OpenCV’s (https://opencv.org/) cv2.adaptiveThreshold function to perform adaptive thresholding. This function binarizes the individual channels by setting each pixel to 1 if its intensity is above the Gaussian weighted mean of its vicinity (here, a window of size 201 × 201) plus a constant *C* (here, the standard deviation of the intensity values across the current protein channel). We then transformed the binarized images into floating point protein abundance matrices *A* by setting the abundance *A*(*c*, *p*) of protein *p* in cell *c* to the fraction of positive binarized pixels within the segment corresponding to *c*.

### Cell-type assignment

Since only few exemplars of the MELC imaging system exist, automated cell-type annotation tools for our data do not exist. Moreover, initial tests showed that cell-type annotation tools developed for data generated by other multiplexing platforms are not applicable for our data. We therefore developed a simple rule-based cell-type annotation workflow, making use of single-cell RNA sequencing (scRNA-seq) data specific to skin tissue from HPA as reference. Specifically, we downloaded normalized gene expression values *X*(*C*, *g*), where *C* denotes cell clusters provided in HPA and *g* denotes genes. Moreover, we made use of HPA’s cell-type annotations *σ*(*C*) ∈ Σ, where Σ is the set of cell types for skin tissue used by HPA. Since, in HPA, there are cell types to which several clusters are assigned, we computed cell-type-specific gene expression values

1 $$X(t,g)={\left(\sum _{C:\sigma (C) = t}| C| \right)}^{-1}\cdot \sum _{C:\sigma (C)=t}| C| \cdot X(C,g)$$

via weighted mean aggregation, for all pairs of cell types *t* and genes *g*.

Let ${\mathcal{C}}$ be the set of all cells contained in any of the samples for any of the three conditions CTCL, PSO, and AD, ${\mathcal{G}}$ be the set of all genes encoding the measured proteins, and *A*(*c*, *g*) be the abundance of the protein encoded by *g* in the cell *c*. Our cell-type assignment workflow iteratively assigns cell types *t*^⋆^ ∈ Σ to a subset ${{\mathcal{C}}}_{{t}^{\star }}\subseteq {\mathcal{C}}$ of the cells. Upon assigning the cell type *t*^⋆^ to the cells contained in ${{\mathcal{C}}}_{{t}^{\star }}$, ${\mathcal{C}}$ and Σ are updated as ${\mathcal{C}}\leftarrow {\mathcal{C}}\setminus {{\mathcal{C}}}_{{t}^{\star }}$ and Σ ← Σ⧹{*t*^⋆^}, respectively. The process stops when either ${\mathcal{C}}={{\emptyset}}$, $\Sigma ={{\emptyset}}$, or there are no good genes (defined below). In the latter two cases, the remaining cells in ${\mathcal{C}}$ are assigned the cell-type label “unknown”.

To find *t*^⋆^ and ${{\mathcal{C}}}_{{t}^{\star }}$ within one iteration, we computed HPA-based spread scores

2 $$s(t,g)=X(t,g)-\max \{X({t}^{{\prime} },g)| {t}^{{\prime} }\in \Sigma \setminus \{t\}\}$$

for all pairs of not yet assigned cell types *t* ∈ Σ and genes $g\in {\mathcal{G}}$ and sorted the pairs in decreasing order of *s*(*t*, *g*), leading to a sorted list *L*. Genes *g* with large spread scores *s*(*t*, *g*) are potential marker genes for the cell type *t*, based on the scRNA-seq data in HPA. Next, we fit a bimodal Gaussian mixture models to the vectors of protein abundances ${(A(c,g))}_{c\in {\mathcal{C}}}$ for the not yet assigned cells (using scikit-learns’s GaussianMixture class) and all genes $g\in {\mathcal{G}}$ and assessed if the obtained distributions are indeed bimodal. For this, we checked the condition

3 $${\mu }_{0}(g)+1.96\cdot {\sigma }_{0}(g)\, <\, {\mu }_{1}(g)-1.96\cdot {\sigma }_{1}(g),$$

where *μ*_0_(*g*) and *σ*_0_(*g*) are the mean and standard deviation values for the mode with the lower mean and *μ*_1_(*g*) and *σ*_1_(*g*) are the mean and standard deviation values for the mode with the higher mean. Genes for which the condition holds are called “good”. When we found at least one good gene, we picked the first pair (*t*^⋆^, *g*^⋆^) from the sorted list *L* for which *g*^⋆^ is good. Then, we assigned the cell type *t*^⋆^ to the cells ${{\mathcal{C}}}_{{t}^{\star }}=\{c\in {\mathcal{C}}| A(c,{g}^{\star })\, >\, {\mu }_{1}({g}^{\star })-1.96\cdot {\sigma }_{1}({g}^{\star })\}$ and continued with the next iteration of our cell-type assignment protocol. When no good gene was found (for our data, this happened when “granulocytes” was the only cell-type label left in Σ), we stopped the protocol.

The resulting rule-based cell-type assignment tree is shown in Supplementary Fig. 1. Heatmaps showing the corresponding (A) cell-type-averaged gene expression values from HPA and (B) protein abundances from our data, are shown in Supplementary Fig. 11. In the following, we let *T* denote the set of used cell-type labels (in our case: the label “unknown” and all labels contained in Σ except “granulocytes”) and let $\lambda :{\mathcal{C}}\to T$ denote the constructed cell-type label function.

### Quantification of spatial tissue heterogeneity with SHouT

SHouT starts by computing sample-specific spatial neighborhood graphs *G* = (*V*, *E*, *λ*_*V*_) from the pre-processed imaging data, where $V\subseteq {\mathcal{C}}$ is the set of cells for the sample under consideration, the set *E* contains an edge $c{c}^{{\prime} }$ for two cells $c,{c}^{{\prime} }\in V$ if *c* and ${c}^{{\prime} }$ are spatially adjacent (computed with Squidpy’s spatial_neighbors function with the parameter delaunay set to True), and *λ*_*V*_ denotes the restriction of the cell-type label function *λ* to *V*. We decided to use Delaunay triangulation to construct the spatial graphs instead of other approaches such as *k*-nearest neighbor or distance threshold graphs because the latter approaches require essential hyper-parameters (the number of neighbors *k* and the distance threshold) which are very difficult to select in a principled way. Based on the spatial graphs, SHouT computes two global scores that quantify heterogeneity for the entire graph *G* and three local, cell-specific scores.

The first global score—global (normalized) entropy—is defined as

4 $$H(G)=-\log {(| T| )}^{-1}\cdot \sum _{t\in T}{p}_{G}(t)\cdot \log ({p}_{G}(t))\in [0,1],$$

where *p*_*G*_(*t*) = ∣{*c* ∈ *V*∣*λ*_*V*_(*c*) = *t*}∣/∣*V*∣ is the fraction of cells in *V* that are of type *t*. Large values of *H*(*G*) indicate that cell-type heterogeneity is high for the sample represented by *G*. The second global score—global homophily—is defined as the fraction of edges

5 $$h(G)=| E{|}^{-1}\cdot \sum _{{c}{c}^{{\prime}}\in {E}}[{\lambda}_{V}(c)={\lambda}_{V}({c}^{{\prime} })]\in [0,1]$$

in the spatial graph *G* that connect cells of the same type ([ ⋅ ]: {True, False} → {0, 1} is the Iverson bracket, i.e., [True] = 1 and [False] = 0). Large values of *h*(*G*) indicate that cells tend to be adjacent to cells of the same type in the sample represented by *G*.

In addition to the four global scores, SHouT provides three local scores to quantify heterogeneity within the *r*-hop neighborhood ${N}_{r}(c)=\{{c}^{{\prime} }\in V| {d}_{G}\left.\right(c,{c}^{{\prime} }\le r\}$ of an individual cell *c* ∈ *V*. Here, *r* is a hyper-parameter and ${d}_{G}:V\times V\to {\mathbb{N}}$ is the shortest path distance. The first local score—local (normalized) entropy—is defined as follows:

6 $${H}_{r}(c)=-\log {(| T| )}^{-1}\cdot \sum _{t\in T}{p}_{{N}_{r}(c)}(t)\cdot \log ({p}_{{N}_{r}(c)}(t))\in [0,1]$$

The only difference to its global counterpart is that the cell-type fractions ${p}_{{N}_{r}(c)}(t)=| \{c\in {N}_{r}(c)| {\lambda }_{V}(c)=t\}| /| {N}_{r}(c)|$ are computed only with respect to the cells contained in the *r*-hop neighborhood of *c*. Also the second local score—local homophily—is defined in a similar way as the global version:

7 $${h}_{r}(c)=| {E}_{{N}_{r}(c)}{| }^{-1}\cdot \sum _{{c}^{{\prime} }{c}^{{\prime\prime} }\in {E}_{{N}_{r}(c)}}[{\lambda }_{V}({c}^{{\prime} })={\lambda }_{V}({c}^{{\prime\prime} })]\in [0,1]$$

Here, the difference to the global version is that we only consider the subset of edges ${E}_{{N}_{r}(c)}=\{{c}^{{\prime} }{c}^{{\prime\prime} }\in E| {c}^{{\prime} },{c}^{{\prime\prime} }\in {N}_{r}(c)\}$ that connect two cells contained in the *r*-hop neighborhood of *c*. The last local score—egophily— does not have a global counterpart. It is defined as the fraction

8 $${e}_{r}(c)={p}_{{N}_{r}(c)}({\lambda }_{V}(c))\in [0,1]$$

of cells within the *r*-hop neighborhood of *c* that have the same cell type as *c*. SHouT provides very efficient vectorized implementations of all heterogeneity scores, relying on SciPy’s sparse.csgraph.shortest_path function for fast computation of shortest path distances.

## Supplementary information

Supplementary figures and tables

## Data Availability

The MELC data underlying this study are available on Zenodo: 10.5281/zenodo.11125482. Pre-clustered, skin-specific scRNA-seq data used as a reference for cell-type assignment are available at https://www.proteinatlas.org/download/rna_single_cell_type_tissue.tsv.zip. The corresponding cluster annotations can be obtained at https://www.proteinatlas.org/download/rna_single_cell_cluster_description.tsv.zip.

## Code availability

The code of the SHouT Python package is available at https://github.com/bionetslab/SHouT. Code to reproduce the results reported in this paper is available at https://github.com/bionetslab/ctcl_case_study.
