# Supplementary material for: Spatial cell graph analysis reveals skin tissue organization characteristic for cutaneous T cell lymphoma
Source: NPJ Syst Biol Appl. 2024 Dec 2;10:143. doi: 10.1038/s41540-024-00474-x (PMC11612425; doi:10.1038/s41540-024-00474-x)
Supplement: Supplementary file 1 — Supplementary figures and tables [file 41540_2024_474_MOESM1_ESM.pdf]

Spatial cell graph analysis reveals skin tissue organization characteristic for cutaneous T cell lymphoma

Suryadipto Sarkar, Anna Möller, Anne Hartebrodt, Michael Erdmann, Andreas Baur, Christian Ostalecki, David B. Blumenthal

Supplementary information

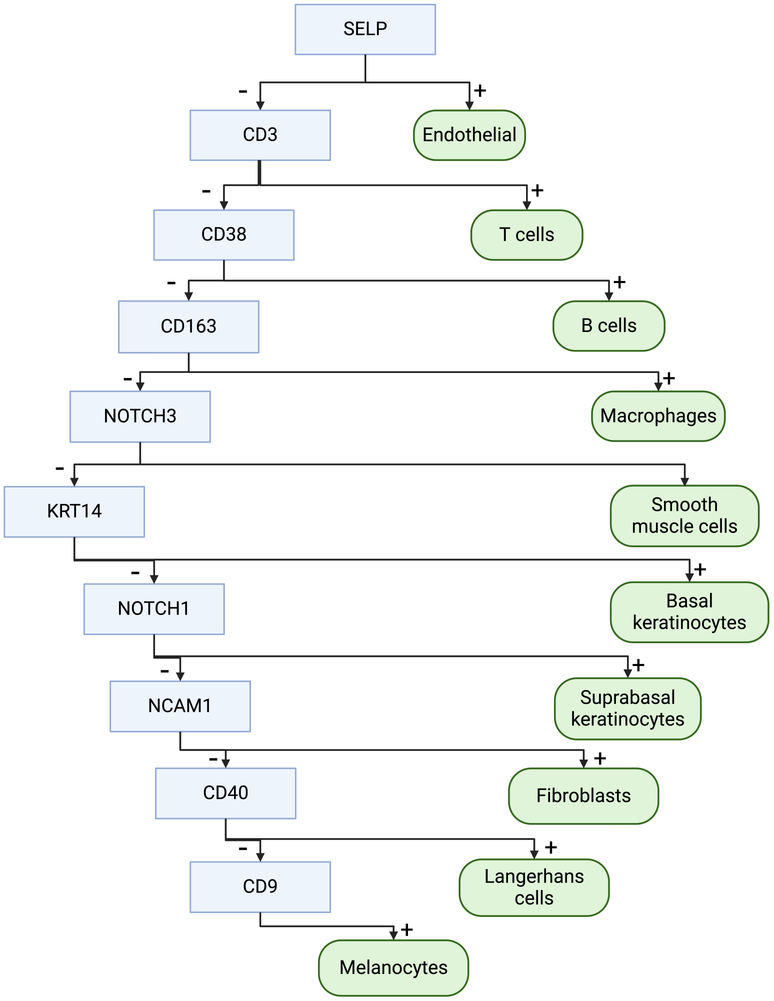

Supplementary Figure 1. Decision tree used for rule-based cell type assignment.

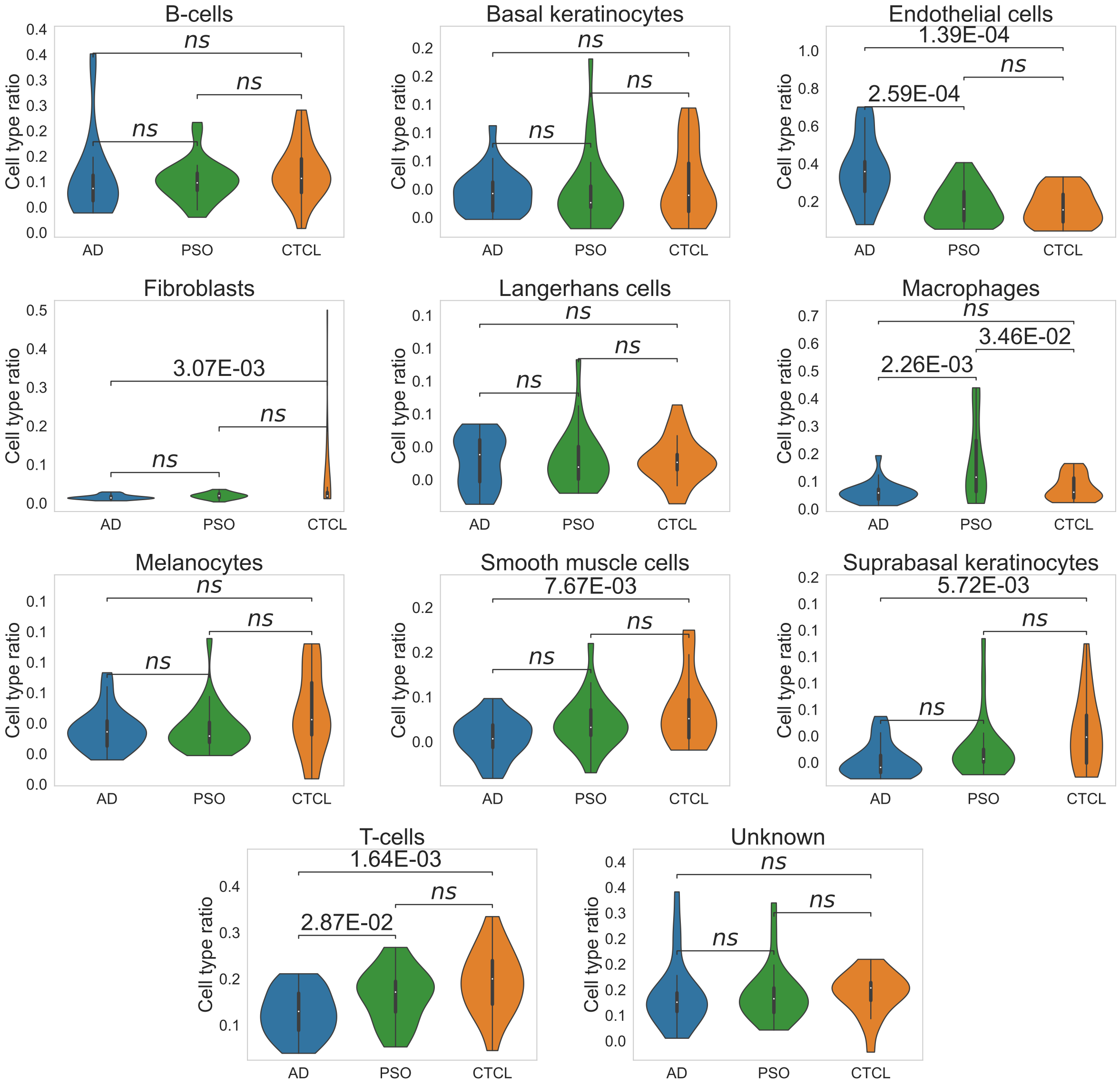

Supplementary Figure 2. Distributions of normalized cell type abundances across all samples, annotated with Bonferonni-corrected MWU *P*-values per condition pair.

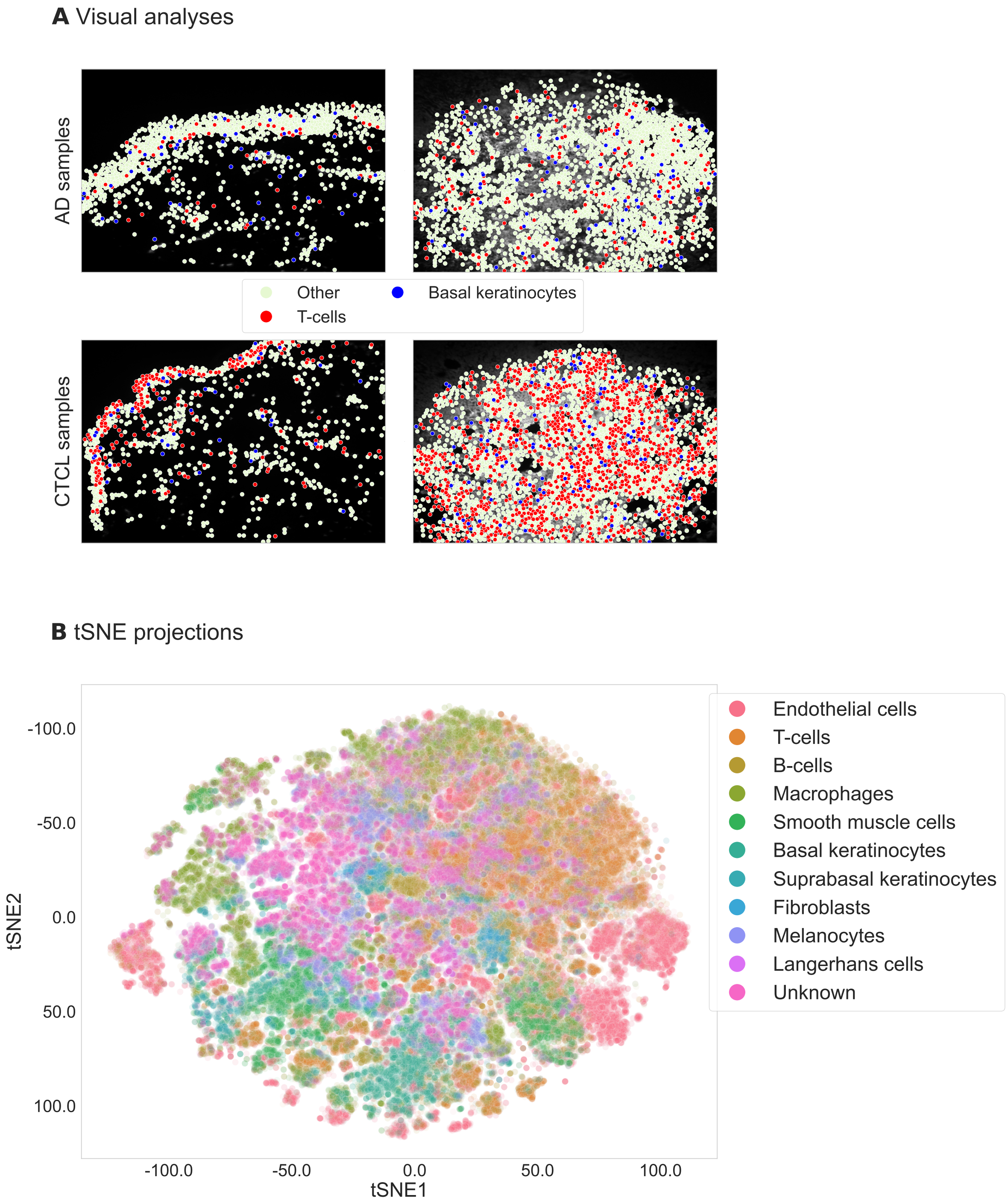

Supplementary Figure 3. A visual overview of the cell type assignment performed on our multi-antigen fluorescence microscopy dataset. (A) A tissue sample from two patients each, having CTCL and AD, with the segmented T-cells, basal keratinocytes and other cell types highlighted in different colors. (B) A scatter plot of the first two t-SNE projections of the protein expression data, combined across all 68 tissues, colored by cell type.

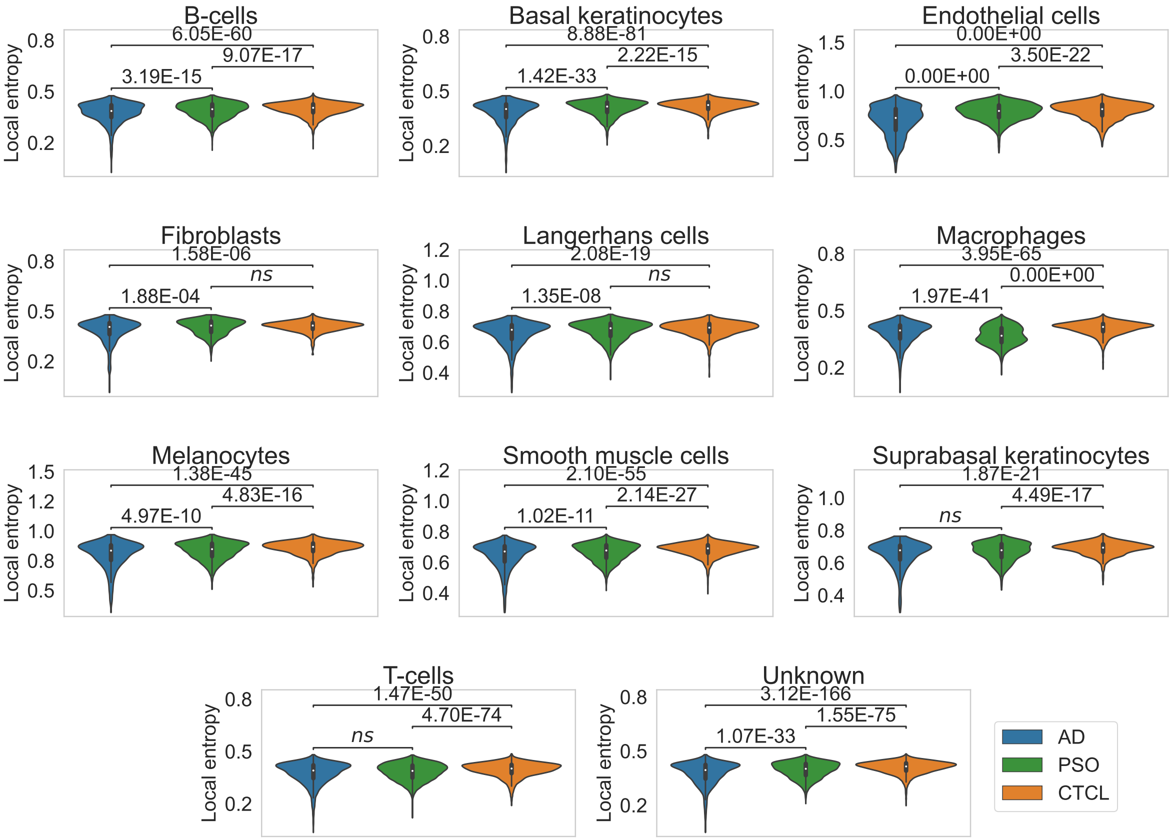

Supplementary Figure 4. Distributions of local entropy with radius $r = 5$, across all samples, annotated with Bonferonni-corrected MWU P-values per condition pair.

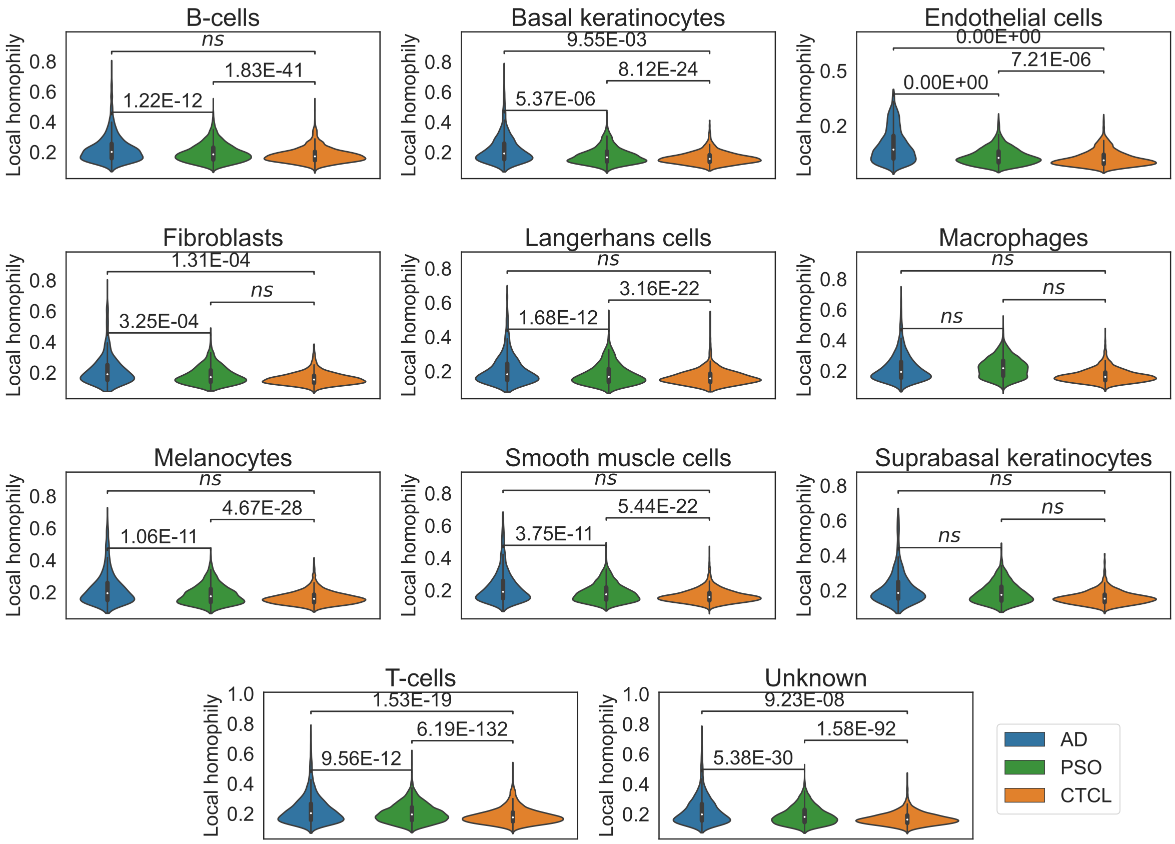

Supplementary Figure 5. Distributions of local homophily with radius $r = 5$, across all samples, annotated with Bonferonni-corrected MWU P-values per condition pair.

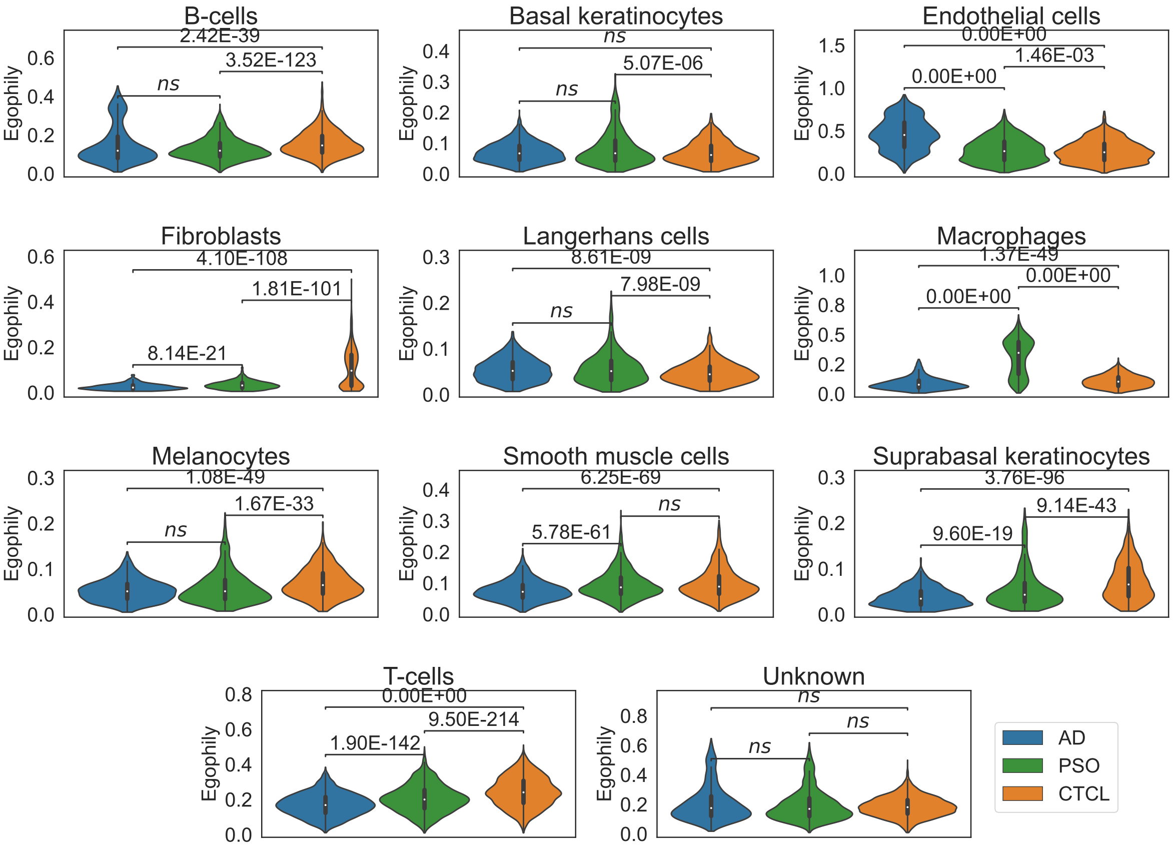

Supplementary Figure 6. Distributions of egophily with radius $r = 5$, across all samples, annotated with Bonferonni-corrected MWU P-values per condition pair.

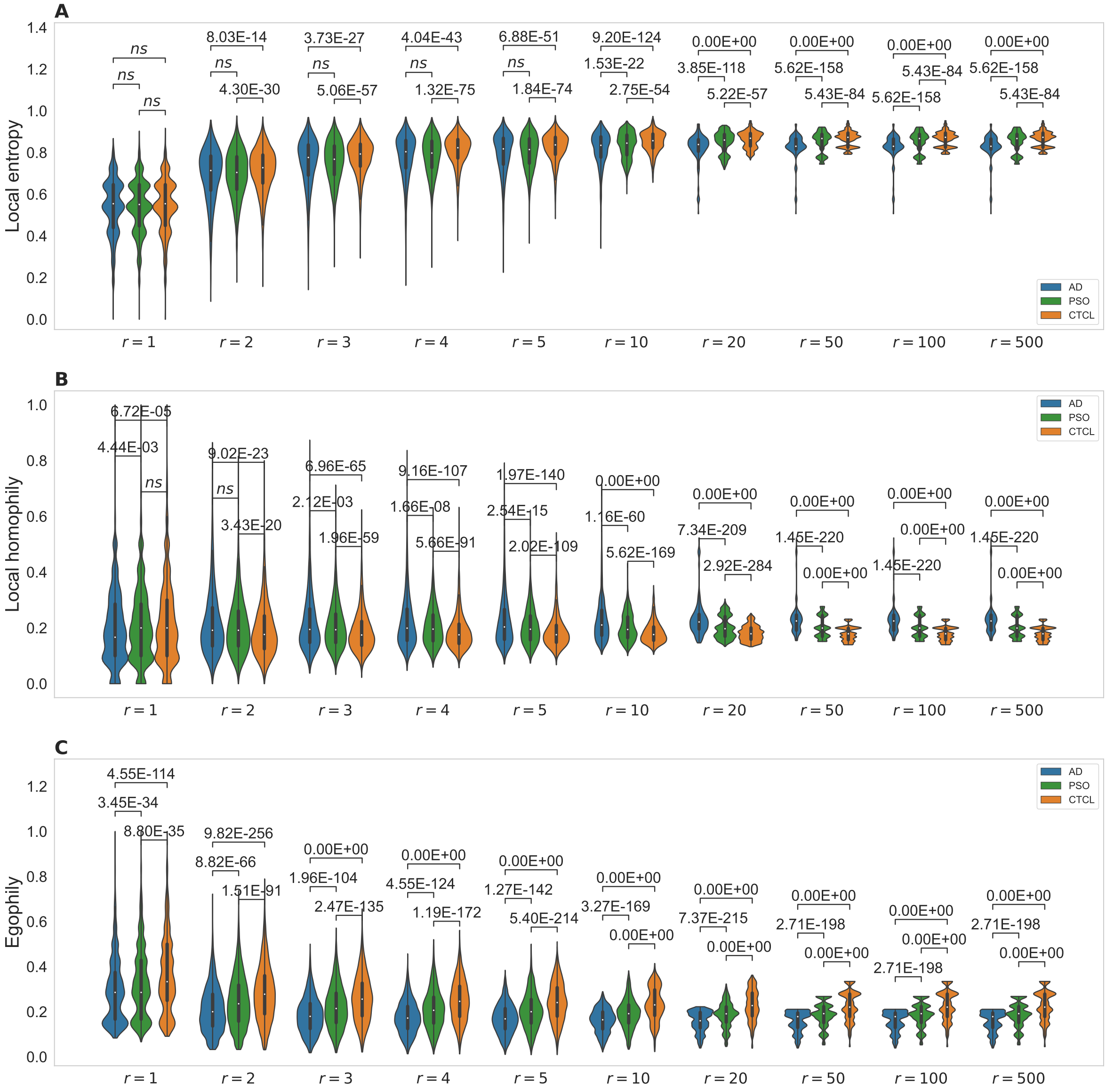

Supplementary Figure 7. Distributions of SHouT local heterogeneity scores (namely, local entropy, local homophily and egophily) across radii $r \in \{1, 2, 3, 4, 5, 10, 20, 50, 100, 500\}$ in T cell neighborhoods as shown in Figure 3 in the main document, as obtained from Delaunay graph. Figure 3 in the main document only contains scores obtained from the Delaunay graph for radii $r \in \{1, 5, 100\}$.

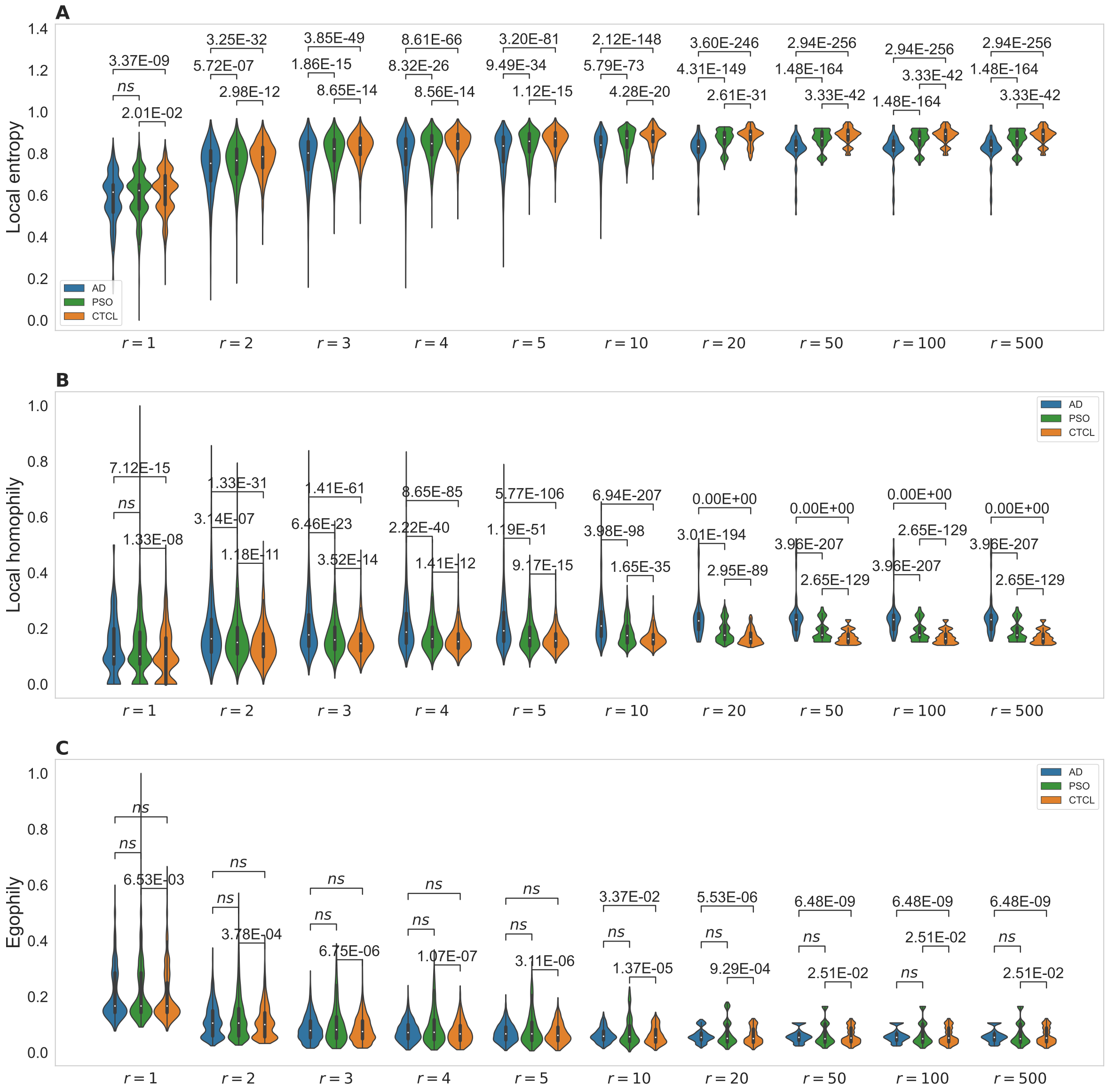

Supplementary Figure 8. Distributions of SHouT local heterogeneity scores (namely, local entropy, local homophily and egophily) across radii $r \in \{1, 2, 3, 4, 5, 10, 20, 50, 100, 500\}$ in basal keratinocyte neighborhoods as shown in Figure 3 in the main document, as obtained from Delaunay graph. Figure 3 in the main document only contains scores obtained from the Delaunay graph for radii $r \in \{1, 5, 100\}$.

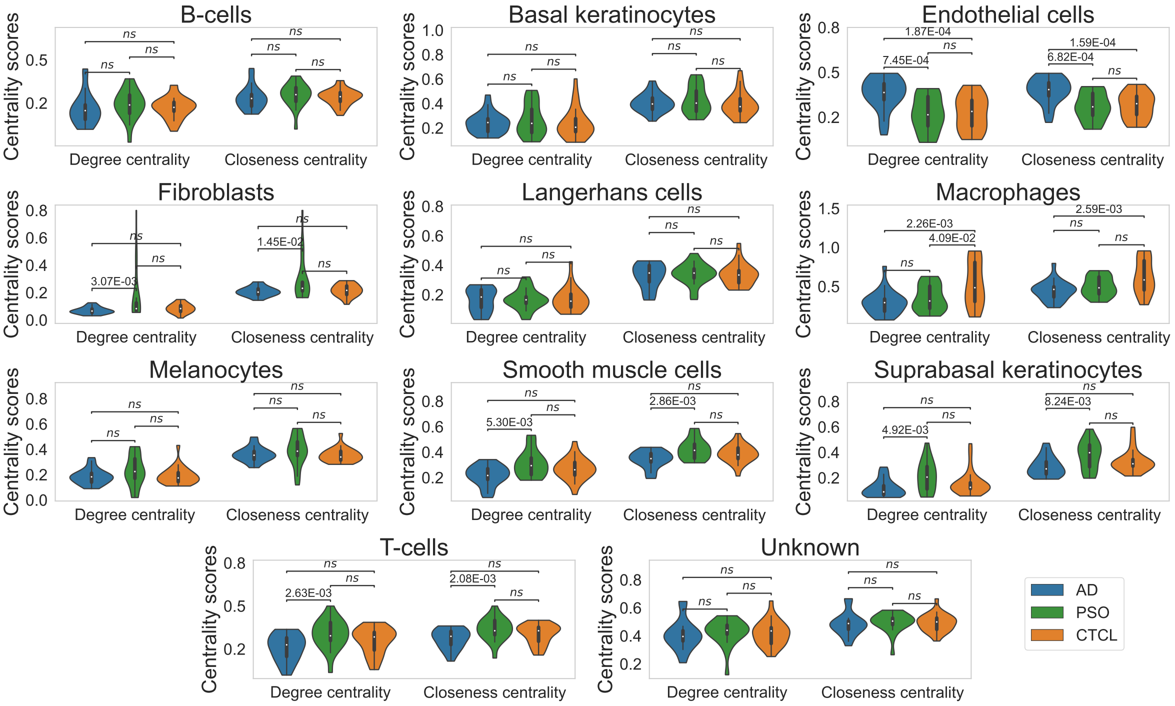

Supplementary Figure 9. Distributions of degree and closeness centrality scores across all samples, annotated with Bonferonni-corrected MWU P-values per condition pair.

A T cells

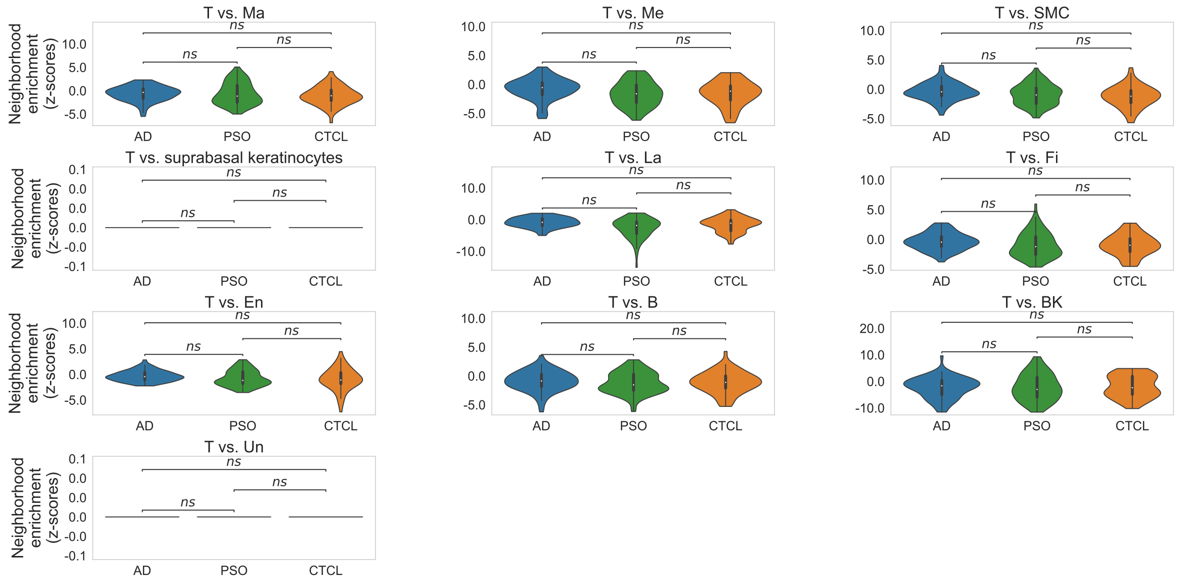

B: B-cells **BK**: Basal keratinocytes **En**: Endothelial cells **Fi**: Fibroblasts
La: Langerhans cells **Ma**: Macrophages **Me**: Melanocytes **SMC**: Smooth muscle cells
SK: Suprabasal keratinocytes **T**: T cells **Un**: Unknown

B Basal keratinocytes

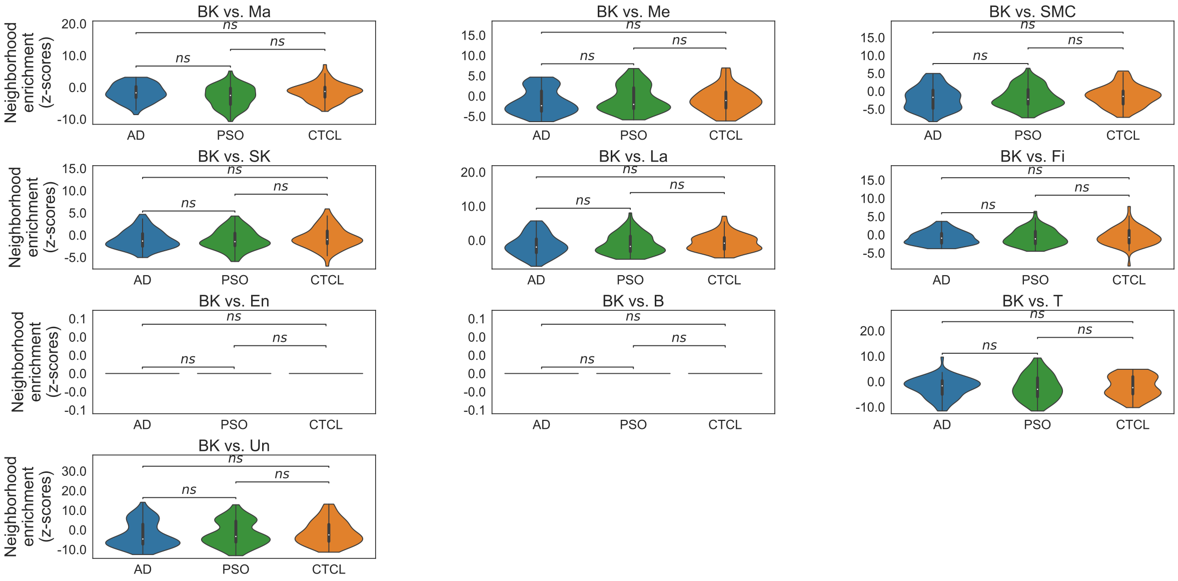

Supplementary Figure 10. Distributions of neighborhood enrichment between (A) T cells and (B) Basal keratinocytes, and each other cell type, across all samples, annotated with Bonferonni-corrected MWU *P*-values per condition pair.

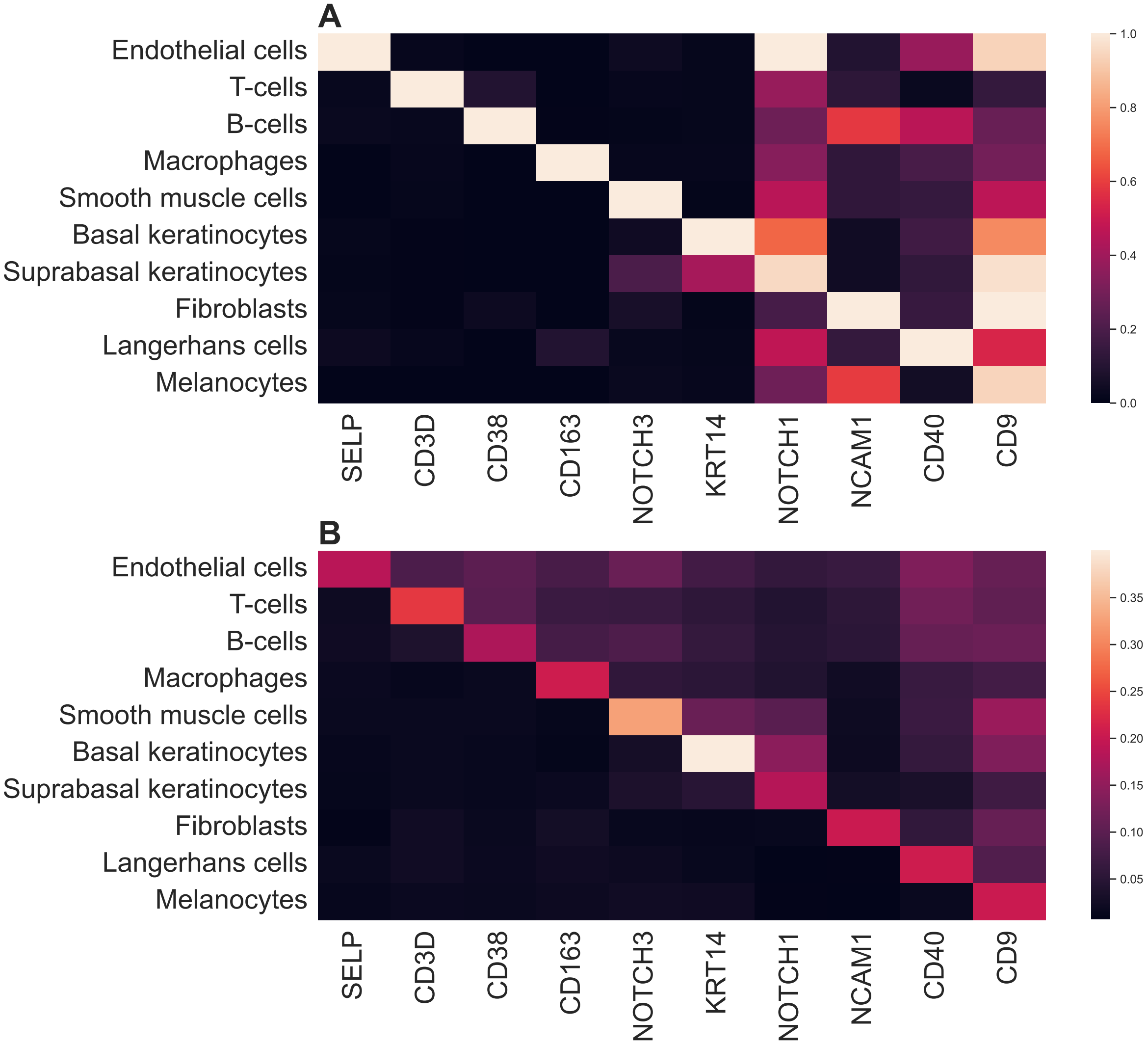

Supplementary Figure 11. Heatmaps showing the (A) cell type-averaged gene expression values from HPA, and (B) protein abundances from our data for the marker proteins used for cell type assignment.

Supplementary Table 1. Marker panel used for this study and mapping to gene names used for cell type assignment based on reference data from HPA. Since unique mapping was not possible for HLA-ABC and HLA-DR, we ran the cell type assignment approach multiple times with different combinations of possible gene name maps. Since no HLA genes were used as marker genes (see Supplementary Figure 1), all runs led to identical results.

Original marker name	Mapped marker(s) for cell type assignment with reference data from HPA
Propodium iodide	–
CD11a	ITGAL
CD11c	ITGAX
CD14	CD14
CD163	CD163
CD205	LY75
CD206	MRC1
CD24	CD24
CD25	IL2RA
CD29	ITGB1
CD3	CD3
CD36	CD36
CD38	CD38
CD4	CD4
CD40	CD40
CD44	CD44
CD45	PTPRC
CD52	CD52
CD54	ICAM1
CD55	CD55
CD56	NCAM1
CD6	CD6
CD62P	SELP
CD63	CD63
CD68	CD68
CD69	CD69
CD8	CD8
CD9	CD9
CD95	FAS
Cytokeratin-14	KRT14
HLA-ABC	HLA-A or HLA-B or HLA-C
HLA-DR	HLA-DRA or HLA-DRB1 or HLA-DRB3 or HLA-DRB4 or HLA-DRB5
Notch-1	NOTCH1
Notch-3	NOTCH3
PPARgamma	PPARG
beta-Catenin	CTNNB1
